# Prolonged *in vivo* expression and anti-tumor response of DNA-based anti-HER2 antibodies

**DOI:** 10.18632/oncotarget.24426

**Published:** 2018-02-06

**Authors:** Kevin Hollevoet, Elien De Smidt, Nick Geukens, Paul Declerck

**Affiliations:** ^1^ Laboratory for Therapeutic and Diagnostic Antibodies, KU Leuven – University of Leuven, Leuven B-3000, Belgium; ^2^ PharmAbs, the KU Leuven Antibody Center – University of Leuven, Leuven B-3000, Belgium

**Keywords:** antibody gene transfer, breast cancer, electroporation, plasmid DNA, trastuzumab

## Abstract

Antibody gene transfer presents an appealing alternative to conventional antibody protein therapy. This pre-clinical study evaluates the impact of various parameters on the pharmacokinetics and efficacy of *in vivo* expressed DNA-based anti-HER2 monoclonal antibodies (mAbs), newly engineered and delivered via intramuscular electrotransfer in mice. Plasma concentrations of trastuzumab and 4D5, its murine IgG1 equivalent, peaked on average between 1–15 µg/ml, depending on the administration and configuration of the encoding plasmid DNA (pDNA). A dual expression cassette system outperformed a single 2A-based cassette, and the CAG promoter was superior to a muscle-specific ΔUSE-based promoter. A ‘gene therapy-compatible’ Gene Transport Unit (gtGTU, FIT Biotech), a plasmid backbone that co-encodes viral elements, failed to improve *in vivo* reporter and mAb expression compared to a conventional plasmid. In BALB/c mice, trastuzumab detection was lost within two weeks after pDNA administration due to anti-drug antibodies. This host immune response was addressed by expressing trastuzumab in immune-compromised mice, or by gene transfer of murine 4D5 in BALB/c mice. Both approaches maintained single-digit µg/ml mAb concentrations for at least six to nine months, and allowed to boost mAb expression over time by pDNA re-dosing. In a breast cancer mouse model, prophylactic and therapeutic DNA-based trastuzumab or 4D5 led to complete tumor regressions, thereby rivalling with the administration of milligrams of mAb protein. In conclusion, our study demonstrates proof of concept for antibody gene transfer in cancer, provides critical insights in the engineering and application of DNA-based antibodies, and serves to advance this modality in oncology and beyond.

## INTRODUCTION

Recombinant monoclonal antibodies (mAbs) are one of today’s most successful therapeutic classes in oncology. A wider accessibility and implementation, however, is hampered by the high production cost and prolonged need for frequent administration. The surge in more effective but costly mAb combination therapies further adds to the financial burden. These issues highlight the need for innovations in conventional mAb production and administration.

*In vivo* antibody gene transfer seeks to administer the mAb-encoding nucleotide sequence, rather than the mAb protein. This allows the site of administration, e.g. the muscle, to produce the therapeutic in a cost- and labor-effective manner for a prolonged period of time. Applied expression platforms, mostly in a pre-clinical setting, include viral vectors, plasmid DNA (pDNA), and, more recently, mRNA [1]. Each of these expression platforms comes with a set of challenges. Our vehicle of interest, pDNA, generally gives a lower transgene expression than viral vectors, but presents less concerns in terms of immunogenicity, biosafety and payload capacity. Compared to pDNA, mRNA typically leads to a more rapid and higher expression, but for a shorter duration of time [1].

Only few studies have evaluated DNA-mediated antibody gene transfer in cancer [1], and it is uncertain whether sufficiently high and prolonged systemic titers can be attained to e.g. impact solid tumors. Different approaches are available to anticipate these concerns. The inefficient *in vivo* transfection of pDNA can be dramatically improved by electroporation, a methodology that has shown to be safe and tolerable for pre-clinical and clinical application [2, 3]. Other factors specifically relate to enhanced construct engineering, and include the choice of promoter, expression cassette configuration, and plasmid backbone – all underexplored in the context of antibody gene transfer.

The current pre-clinical study aims to build proof of concept for intramuscular antibody gene electrotransfer in oncology, and evaluate the impact of several relevant parameters on the pharmacokinetics (PK) and efficacy of DNA-based antibodies. Initial assessment of pDNA electrotransfer and design was done using a novel triple-reporter construct. The anti-HER2 trastuzumab served as a model for antibody gene transfer, as it presents a well-characterized and effective mAb in the field of oncology [4] for which a murine equivalent, i.e. 4D5 [5], and clinical reference material are available.

## RESULTS

### *In vivo* validation of a triple-reporter plasmid

**Figure 1:**
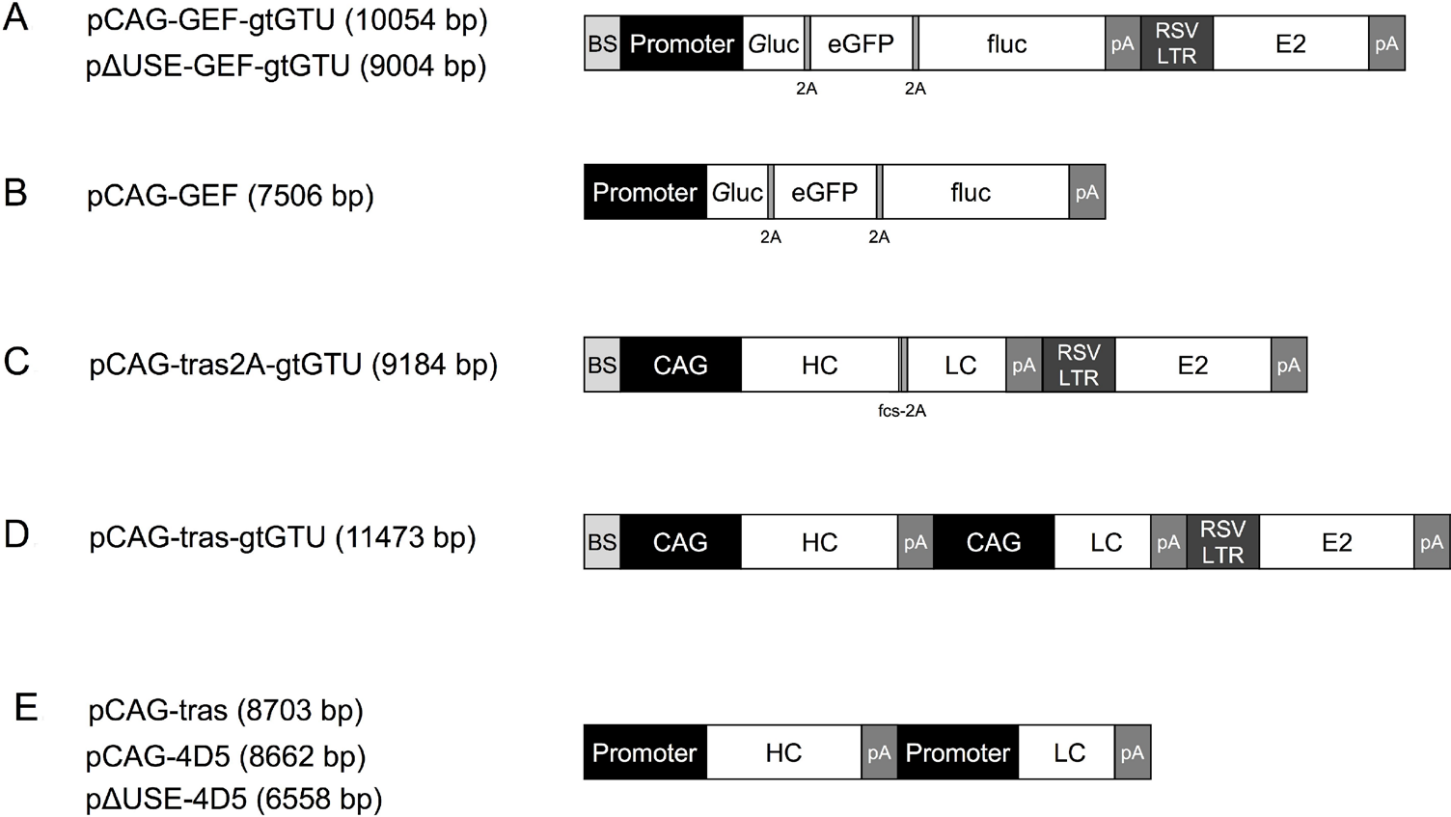
Schematic reporter- or antibody-encoding plasmid configurations Abbreviations; BS: E2 binding sites, eGFP: enhanced green fluorescent protein, fcs: furin cleavage site, fluc: firefly luciferase, *G*luc: *Gaussia* luciferase, gtGTU: gene therapy-compatible Gene Transport Unit, HC: heavy chain, LC: light chain, pA: poly A, RSV LTR: Rous sarcoma virus 5′ long terminal repeat promoter.

**Figure 2:**
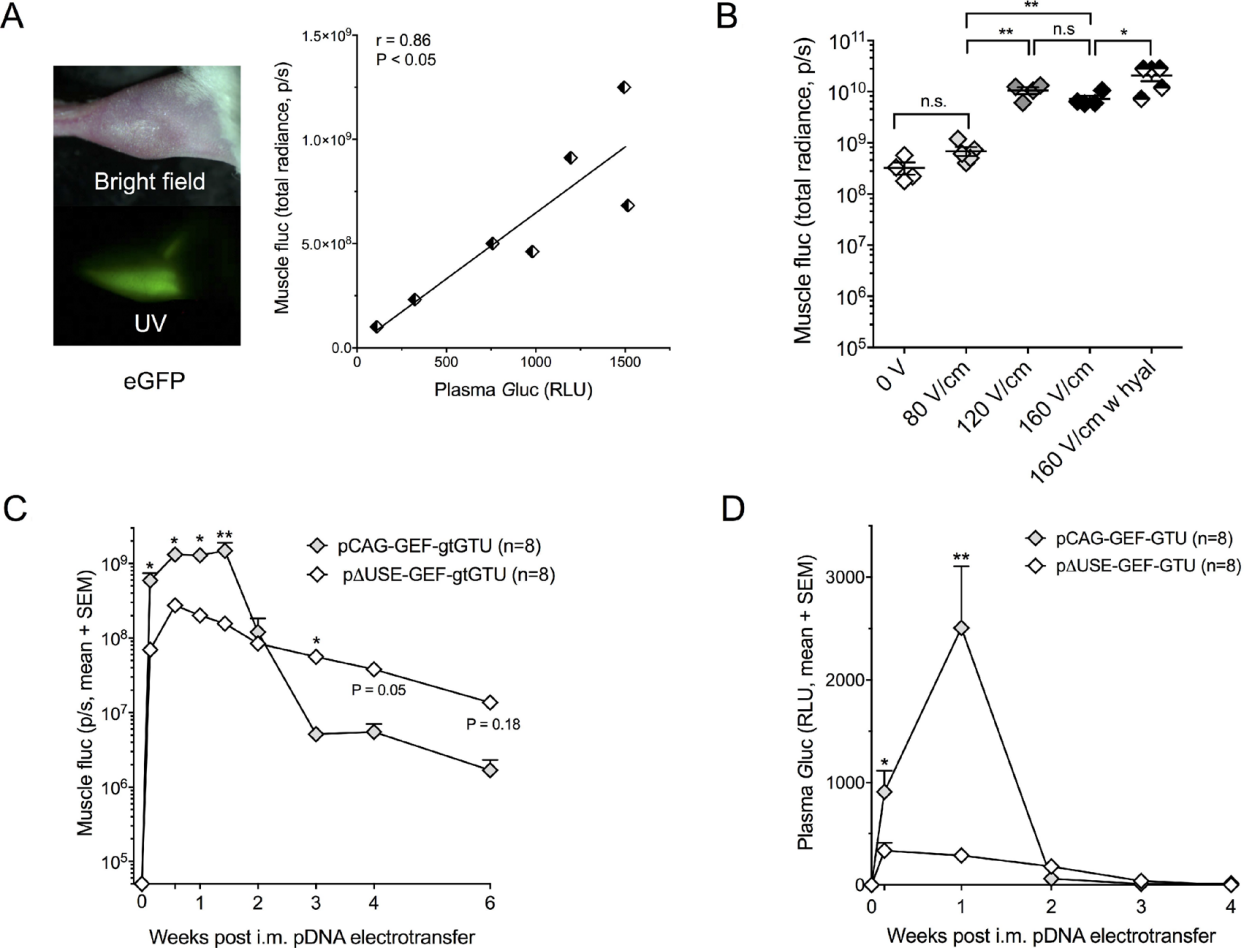
Intramuscular reporter gene transfer in BALB/c mice (**A**) Expression of enhanced green fluorescent protein (eGFP), *Gaussia* luciferase (*G*luc) and firefly luciferase (fluc) one day after intramuscular (i.m.) pCAG-GEF-gtGTU electrotransfer. (**B**) Impact of applied electrical field and hyaluronidase (hyal) pretreatment on muscle fluc expression one day after i.m. pCAG-GEF-gtGTU. ^*^P < 0.05, ^**^P < 0.005. (**C–D**) Intramuscular fluc (C) and plasma *G*luc (D) resulting from the CAG promoter and the muscle-specific ΔUSE promoter. ^*^P < 0.05, ^**^P < 0.005. Abbreviations; gtGTU: gene therapy-compatible Gene Transport Unit, n.s.: not significant, p/s: photons per second, RLU: relative light unit, UV: ultraviolet.

We established a triple-reporter expression cassette, in which a CAG promoter drives the expression of *Gaussia* luciferase (*G*luc), enhanced green fluorescent protein (eGFP) and firefly luciferase 2 (fluc) (CAG-GEF), each separated from one another using a self-cleaving foot-and-mouth-disease virus (FMDV) 2A peptide [6]. *G*luc, a 20-kDa protein, is secreted extracellularly, while eGFP and fluc remain intracellularly. eGFP allows for microscopic and histological analyses, while *G*luc and fluc are detected via bioluminescent imaging of plasma and mice, respectively. The expression cassette was cloned into a ‘gene therapy-compatible’ Gene Transport Unit (gtGTU), a plasmid backbone developed by FIT Biotech (Finland) that co-encodes the nuclear anchoring E2 protein and harbors multimeric E2 binding sites (pCAG-GEF-gtGTU, Figure 1A) [7, 8]. After verifying *in vitro* expression (data not shown), *in vivo* functionality was evaluated via intramuscular electrotransfer in BALB/c mice (*n* = 7). The applied electroporation protocol consisted of three series of four 20 ms square-wave pulses of 120 V/cm with a 50 ms interval between the pulses and polarity switching after two of the four pulses. One day post pDNA administration, all three reporters were detected (Figure 2A), with plasma *G*luc and muscle fluc being highly correlated (*r* = 0.86, *P* < 0.05). Whole-mice bio-luminescent imaging confirmed that fluc expression was restricted to the electroporated muscle site (data not shown). Given the less quantitative nature of eGFP, this readout was not included in current and subsequent analyses. These findings demonstrated that pCAG-GEF-gtGTU is functional *in vivo*, and allows for simultaneous detection of both localized and secreted reporters.

### Impact of electrical field strength and hyaluronidase pretreatment

In addition to the above applied electroporation protocol, two additional field strengths (80 V/cm and 160 V/cm) were evaluated in BALB/c mice, while maintaining the other pulse parameters. Muscle fluc, one day after pCAG-GEF-gtGTU electrotransfer, served as readout (Figure 2B). A field strength of 80 V/cm did not increase fluc expression compared to no electroporation (*P* = 0.986), unlike 120 V/cm and 160 V/cm (*P* < 0.005). Increasing the field strength from 120 to 160 V/cm failed to further improve fluc (*P* = 0.994). These observations were maintained throughout a three-week follow-up (data not shown). Increasing the number of pulse series from three to six did not impact fluc expression. Injecting the muscle with 40 µl of 0.4 U/µl hyaluronidase, an enzyme that degrades hyaluronic acid and increases tissue permeability, prior to electroporation enhanced fluc expression by 3.2-fold (*P* < 0.05) (Figure 2B). Increasing the dose of hyaluronidase from 0.4 to 4 U/µl did not increase fluc (data not shown). Following these results, the three series of 120 V/cm pulses were maintained in subsequent experiments, unless stated otherwise. The use of hyaluronidase was restricted to the antibody gene transfer work.

### Added value of a muscle-specific promoter for reporter gene transfer

Using plasma *G*luc and muscle fluc as readouts, the ubiquitous CAG promoter was compared with a muscle-specific promoter in BALB/c mice. The latter is based on three copies of a modified UpStream Enhancer (ΔUSE) of the human slow troponin I gene, linked to the gene’s minimal promoter [9] (pΔUSE-GEF-gtGTU, Figure 1A). Following intramuscular electrotransfer of equimolar pDNA amounts, CAG outperformed ΔUSE in the first seven to ten days for both reporters (Figure 2C–2D). Plasma *G*luc was not detected beyond two weeks (Figure 2D), whereas muscle fluc expression remained detectable for at least six weeks. During follow-up, CAG fluc did decrease more pronounced than ΔUSE fluc, with the latter outperforming CAG beyond day 14 (Figure 2C). These data hinted that a muscle-specific promoter could lead to a more stable prolonged transgene expression.

### Added value of the gtGTU for reporter gene transfer

**Figure 3:**
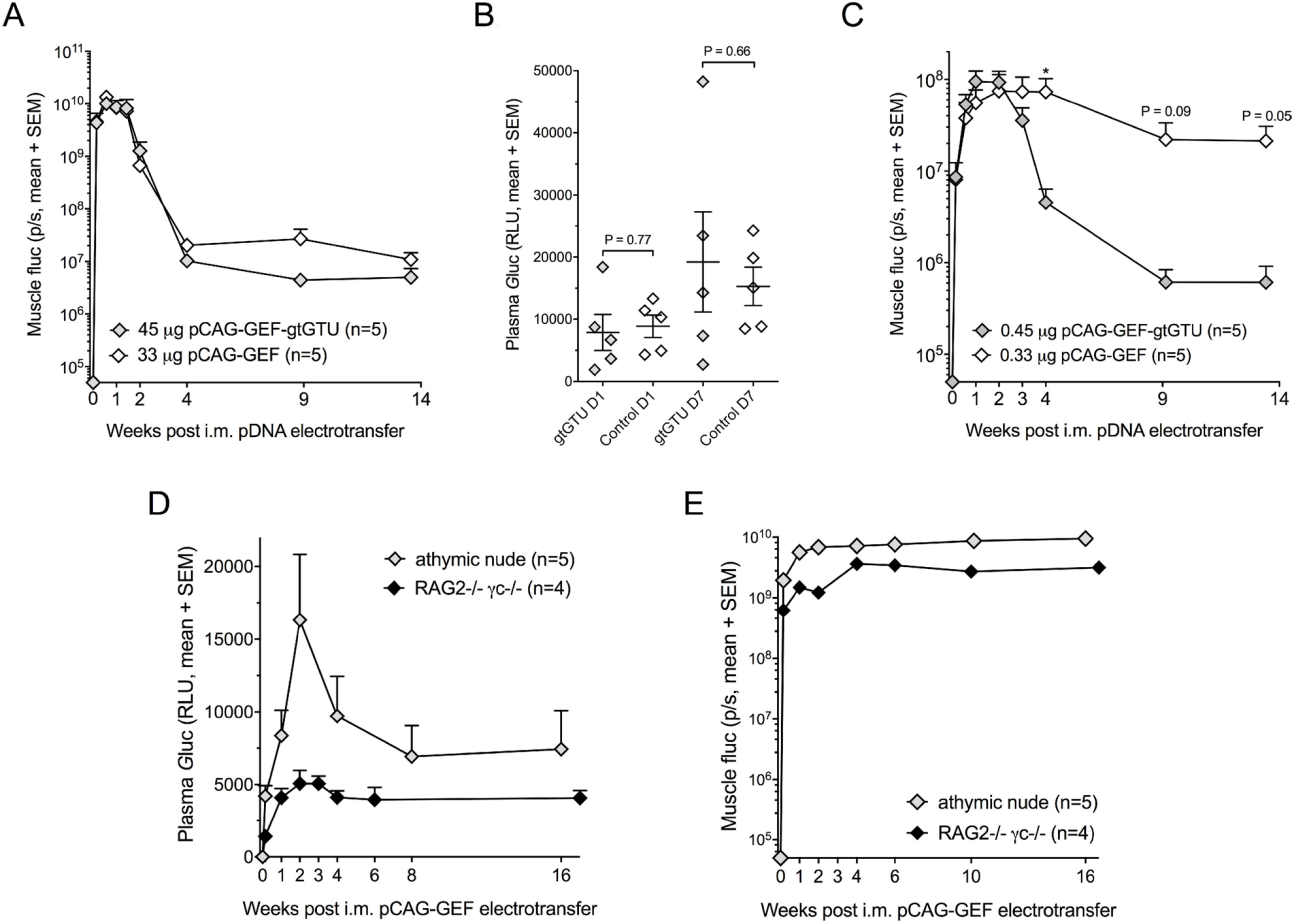
Added value of the gtGTU plasmid for reporter gene transfer (**A–C**) Muscle firefly luciferase (fluc) (A and C) and plasma *Gaussia* luciferase (*G*luc) (B) in BALB/c mice following intramuscular (i.m.) electrotransfer of equimolar amounts of pCAG-GEF-gtGTU or conventional control pCAG-GEF. ^*^P < 0.05. Plasma *G*luc is displayed one and seven days after pDNA electrotransfer. (**D–E**) Muscle fluc (D) and plasma *G*luc (E) in athymic nude and RAG2^*−/−*^γc^*−/−*^ mice. Abbreviations; p/s: photons per second, RLU: relative light unit.

To assess its benefit, gtGTU was compared to a conventional plasmid, identical in design except for the presence of the co-encoded viral elements (pCAG-GEF, Figure 1B). Intramuscular electrotransfer of 45 µg pCAG-GEF-gtGTU and equimolar amounts of pCAG-GEF in BALB/c mice showed a highly similar muscle fluc during the 14-week follow-up (Figure 3A). Similarly, no differences in plasma *G*luc were observed in the first week following pDNA electrotransfer, before the signal was lost (Figure 3B). Both constructs were subsequently compared at 100-fold lower doses. Plasma *G*luc resulting from these low pDNA amounts was not detectable. A dose-response between administered pDNA and muscle fluc was obvious. No difference in fluc expression was present between both plasmids in the first two weeks. Similar as observed with the 100-fold higher dosing, gtGTU fluc decreased three weeks after pDNA electrotransfer (Figure 3C). In contrast, fluc from the conventional plasmid remained stable for a longer period of time. The reason for the more rapid decrease in gtGTU fluc could be linked to a more pronounced immune response, but is beyond the scope of the current study. Due to poor performance, gtGTU use was discontinued in the remaining reporter experiments.

### Reporter gene transfer in immune-compromised mice

To assess the impact of the host immune system on reporter expression, fluc and *G*luc expression following pCAG-GEF intramuscular electrotransfer were evaluated in immune-compromised athymic nude and RAG2^*−/−*^γc^*−/−*^ mice. Although the resulting reporter expressions varied somewhat between each strain, muscle fluc and plasma *G*luc remained highly stable throughout the 16–17 weeks of follow-up (Figure 3D–3E). These data indicated that the decrease of reporter expression in BALB/c mice (Figure 3A) is linked to an immune response against the expressed reporters.

### Pharmacokinetics of DNA-based trastuzumab in BALB/c mice

**Figure 4:**
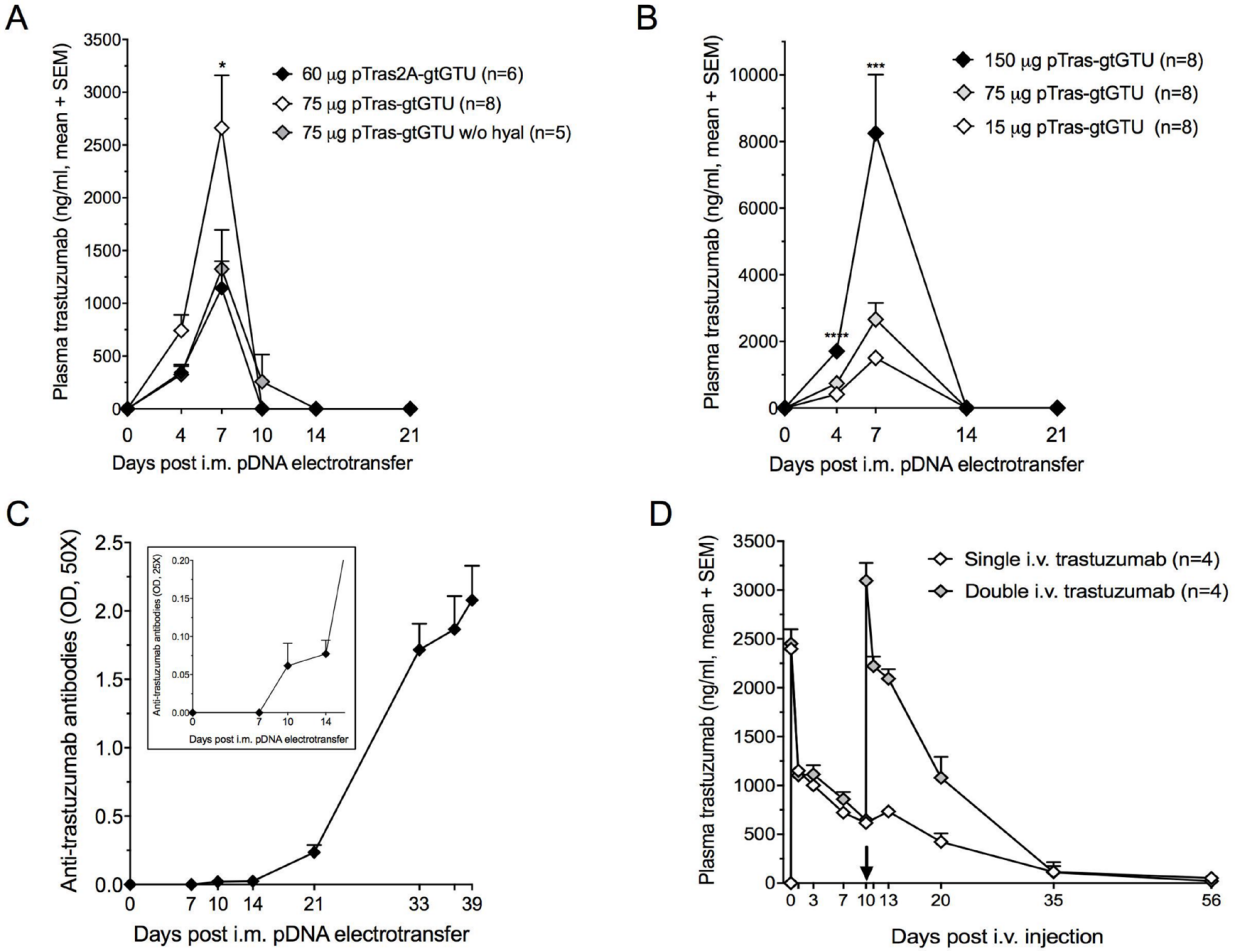
Trastuzumab gene transfer and trastuzumab protein administration in BALB/c mice (**A**) Impact of hyaluronidase (hyal) pretreatment and administered construct configuration (pCAG-tras-gtGTU, a dual antibody cassette system, *vs.* pCAG-tras2A-gtGTU, a single 2A-driven antibody expression cassette) on expressed plasma trastuzumab concentrations (^*^P < 0.05). (**B**) Dose-response between administered pCAG-tras-gtGTU and expressed plasma trastuzumab concentrations. 150 µg pDNA was equally divided across two *tibialis anterior* muscles. 15 µg and 75 µg were injected in one *tibialis anterior* muscle (^***^P < 0.001, ^****^P < 0.0001). (**C**) Anti-trastuzumab antibodies start emerging 10 days after intramuscular (i.m.) electrotransfer of 75 µg pCAG-tras-gtGTU. A second pCAG-tras-gtGTU dose was given 32 days after the first. Presented optical densities (OD) result from 50-fold diluted plasma samples after background subtraction. The figure insert shows the same samples, diluted 25-fold to highlight anti-trastuzumab antibodies in the first two weeks. Data are presented as mean ± standard error of mean. (**D**) Plasma trastuzumab titers resulting from one or two intravenous (i.v.) injections of 0.2 mg/kg trastuzumab. The first plasma sample post injection was collected after one hour. The arrow indicates the timing of the second i.v. trastuzumab injection.

The impact of different variables on the PK of *in vivo* expressed trastuzumab was evaluated in BALB/c mice. First, two novel mAb cassette configurations were engineered. With gtGTU still under evaluation for reporter gene transfer at that time, this plasmid backbone was initially used. In pCAG-tras2A-gtGTU, mAb expression was driven by a single CAG promoter, with the trastuzumab heavy chain (HC) and light chain (LC) separated by a RKRR furin cleavage site and FMDV 2A peptide (Figure 1C) [10, 11]. In pCAG-tras-gtGTU, a dual cassette approach is applied, in which each mAb chain is incorporated into an identical CAG-driven cassette (Figure 1D). When comparing both configurations, the dual cassette outperformed the single cassette (2.3-fold difference at day 7, *P* < 0.05). Second, in agreement with the reporter findings, hyaluronidase injection of the muscle prior to electroporation led to an increased mAb expression (2-fold difference at day 7, *P* < 0.05) (Figure 4A). Based on these findings, the dual cassette configuration and hyaluronidase pretreatment was used in all ensuing experiments. Third, the earlier observed dose-response for reporter pDNA was also obvious for trastuzumab, enabling higher mAb titers via increased doses or targeting multiple muscle sites (Figure 4B). These data demonstrated that plasma trastuzumab expression peaks at µg/ml concentrations, but is lost within two weeks after pDNA electrotransfer, irrespective of the applied variables.

### Antibody immune response against *in vivo* expressed trastuzumab

The loss of trastuzumab in BALB/c mice was found to correlate with the emergence of anti-trastuzumab antibodies in all mice, as early as 10 days after pCAG-tras-gtGTU electrotransfer (Figure 4C). Administration of an additional identical pDNA dose, one month after the first injection, failed to result in any detectable trastuzumab, but rather appeared to increase anti-drug antibody (ADA) titers. Presented data were collected using a drug-sensitive ADA assay, and confirmed with a drug-tolerant assay (data not shown). i.v. injection of 0.2 mg/kg trastuzumab protein (Herceptin, Roche) in naïve BALB/c mice resulted in similar peak levels compared to DNA-based delivery, but overall gave a different picture. The injected trastuzumab remained detectable in plasma for five weeks after injection. An additional i.v. 0.2 mg/kg trastuzumab dose in four out of eight mice, 10 days after the first injection, gave a PK profile highly similar to the first administration (Figure 4D). Anti-trastuzumab antibodies were only detected five weeks after the first i.v. injection in half of the mice (not linked to the number of received i.v. injections) (Supplementary Figure 1A). These findings indicated that ADAs are a critical hurdle to attain prolonged mAb expression, and that DNA-based delivery expedites this immune response compared to conventional protein administration.

### Pharmacokinetics of DNA-based trastuzumab in immune-compromised mice

**Figure 5:**
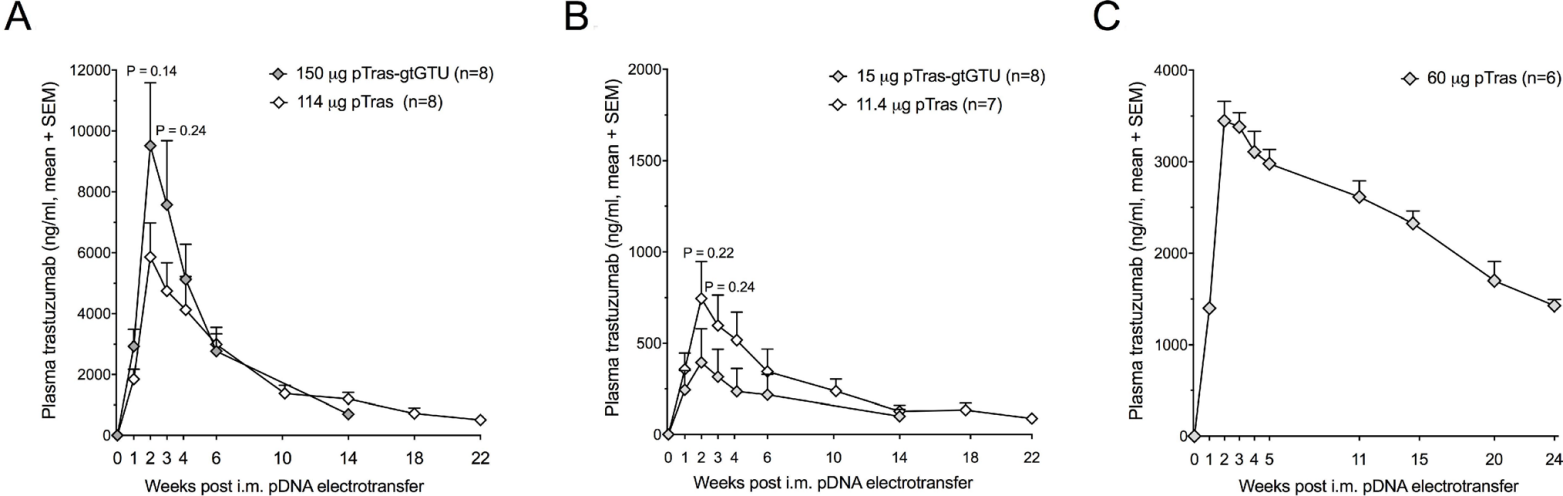
Added value of the gtGTU plasmid for trastuzumab gene transfer in immune-compromised mice (**A**–**B**) Plasma trastuzumab concentrations resulting from intramuscular (i.m.) electrotransfer of equimolar amounts of pCAG-tras-gtGTU and conventional pCAG-tras in athymic nude mice. (**C**) Plasma trastuzumab concentrations following i.m. electrotransfer of pCAG-tras in RAG2^*−/−*γ^c^*−/−*^ mice.

To address the ADA response, trastuzumab gene transfer was evaluated in athymic nude mice. In the meantime, it had become clear that gtGTU provided no benefit for *in vivo* reporter expression. We therefore compared pCAG-tras-gtGTU with conventional pCAG-tras, identical in design except for the E2 gene cassette and E2 binding sites (Figure 1E). At different equimolar pDNA doses, gtGTU failed to show a benefit in trastuzumab expression compared to the conventional plasmid (Figure 5A–5B). For both constructs, plasma trastuzumab peaked two to three weeks post pDNA electrotransfer, and remained detectable for several months. Follow-up of the gtGTU group was discontinued 14 weeks post pDNA administration. The conventional pDNA group continued to be monitored for 22 weeks, with plasma trastuzumab maintaining up to single-digit µg/ml concentrations. Given the lack of benefit, gtGTU use was discontinued in favor of a conventional plasmid in all ensuing experiments. Intramuscular electrotransfer of pCAG-tras in RAG2^*−/−*^γc^*−/−*^ mice, a more immune-compromised strain, demonstrated a similar trastuzumab PK as in nude mice, both in terms of amount and duration of expression (Figure 5C). As expected, no anti-trastuzumab antibodies were detected. These data demonstrated that *in vivo* expression of trastuzumab can be maintained for several months in the absence of a competent host immune system.

### Pharmacokinetics of DNA-based 4D5 in BALB/c mice

**Figure 6:**
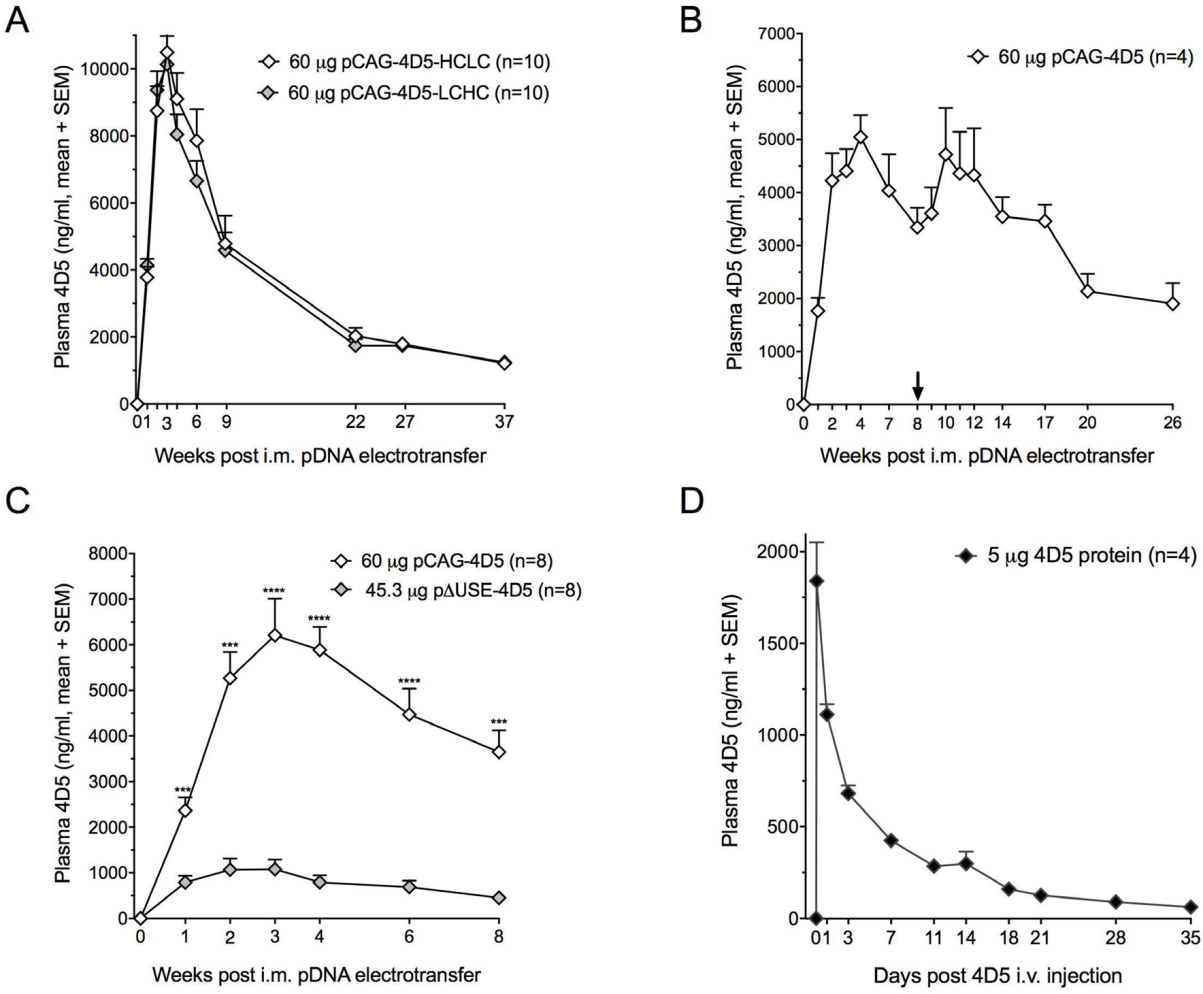
4D5 gene transfer and 4D5 protein administration in BALB/c mice (**A**) Plasma 4D5 concentrations following a single intramuscular (i.m.) electrotransfer of pCAG-4D5-HCLC (heavy chain – light chain order) and pCAG-4D5-LCHC (light chain – high chain order). (**B**) Impact of repeated electrotransfer of 60 µg pCAG-4D5 on plasma 4D5 concentrations. The arrow indicates the timing of the second pCAG-4D5 dose. (**C**) Plasma 4D5 concentrations resulting from i.m. electrotransfer of equimolar amounts of pCAG-4D5 and muscle-specific p∆USE-4D5. ^***^P < 0.001, ^****^P < 0.0001. (**D**) Plasma 4D5 pharmacokinetics of a single intravenous (i.v.) injection of 5 µg purified 4D5. The first plasma sample post injection was collected after one hour.

We subsequently focused on achieving prolonged mAb production in immune-competent BALB/c mice, a more clinically relevant context. This was pursued by gene electrotransfer of 4D5, the murine equivalent of humanized trastuzumab [5]. 4D5 cDNA sequences were cloned into the dual CAG cassette configuration and conventional plasmid that was used for trastuzumab (pCAG-4D5, Figure 1E). Besides sequence origin, the impact of additional construct engineering and pDNA re-dosing on 4D5 PK was assessed. Swapping the order of the HC and LC cassettes (pCAG-4D5-HCLC versus pCAG-4D5-LCHC) demonstrated no difference throughout follow-up. Both constructs enabled high plasma 4D5 titers, peaking after three weeks at approximately 10 µg/ml, and maintained single-digit µg/ml concentrations for at least nine months (Figure 6A). Given the lack of difference, the original HC LC cassette order (Figure 1E) was maintained in subsequent experiments. Throughout follow-up, no anti-4D5 antibodies were detected using either a drug-sensitive or a drug-tolerant ADA assay. Accordingly, a second pCAG-4D5 electrotransfer in the non-transfected *tibialis anterior* of BALB/c mice, eight weeks after the initial dose, resulted in a boost in 4D5 plasma concentrations (Figure 6B). Throughout an eight-week follow-up, CAG enabled five- to six-fold higher 4D5 concentrations compared to the muscle-specific ΔUSE promoter (Figure 6C). This is different from the fluc data (Figure 2C), which could be linked to the absence of immune response against 4D5. As a consequence, the CAG construct was maintained in all subsequent experiments. In contrast to DNA-mediated 4D5 delivery, the PK profile of a single i.v. injection of 5 µg of purified 4D5 in BALB/c mice showed rapid loss of the circulating mAb, with only marginal concentrations remaining three weeks after injection (Figure 6D). These findings illustrated the impact of construct configurations on mAb titers, with species-matched mAb sequences enabling prolonged *in vivo* expression in immune-competent animals.

### Characterization of *in vitro* expressed trastuzumab and 4D5

**Figure 7:**
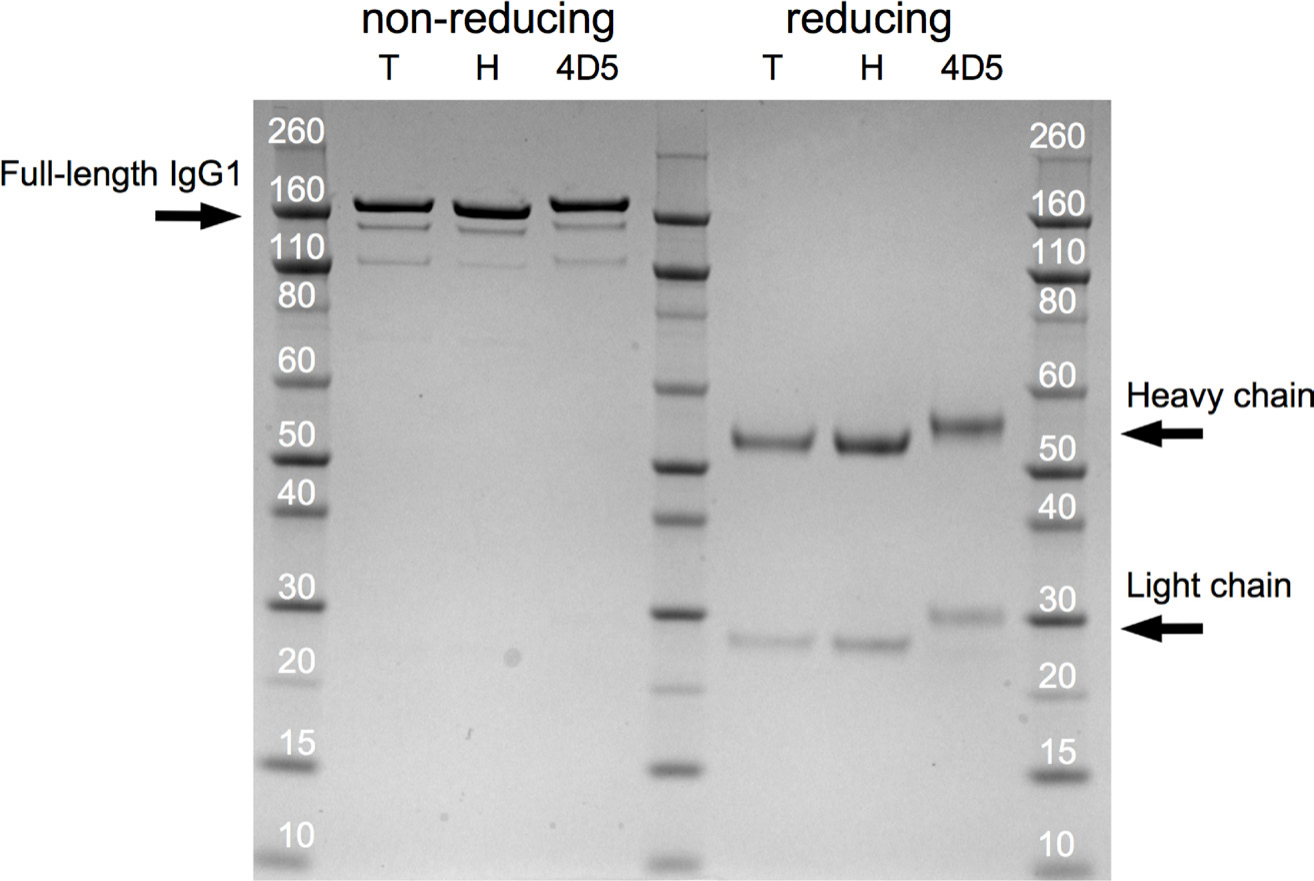
Trastuzumab and 4D5 SDS-PAGE profile Coommasie blue-stained gel following SDS-PAGE of 2.5 µg of in-house expressed and purified trastuzumab (‘T’), clinical-grade trastuzumab (‘H’, Herceptin, Roche) and in-house expressed and purified 4D5 (‘4D5’) under non-reducing and reducing conditions.

*In vitro* expressed and purified trastuzumab and 4D5 demonstrated the expected molecular weight on SDS-PAGE under non-reducing and reducing conditions. ‘In-house’ generated trastuzumab was highly similar to clinical-grade trastuzumab (Herceptin, Roche) (Figure 7). The biological activity of these mAbs was evaluated in a five-day proliferation assay of BT474, a HER2+ human breast cancer cell line. In accordance with the literature [5], 4D5 (IC_50_ = 169.1 ng/ml + 8.7 ng/ml, mean + SEM, *n* = 6) was more active than trastuzumab (IC_50_ = 224.4 ng/ml + 22.1 ng/ml, mean + SEM, *n* = 6, *P* < 0.05). In-house trastuzumab displayed a similar activity to clinical-grade trastuzumab (IC_50_ = 231.9 + 11.4 ng/ml, *n* = 6, *P* = 0.77). These results confirmed that the engineered pCAG-4D5 and pCAG-tras constructs express functional and biologically active anti-HER2 mAbs.

### Efficacy of DNA-based trastuzumab and 4D5 in a breast cancer model

**Figure 8:**
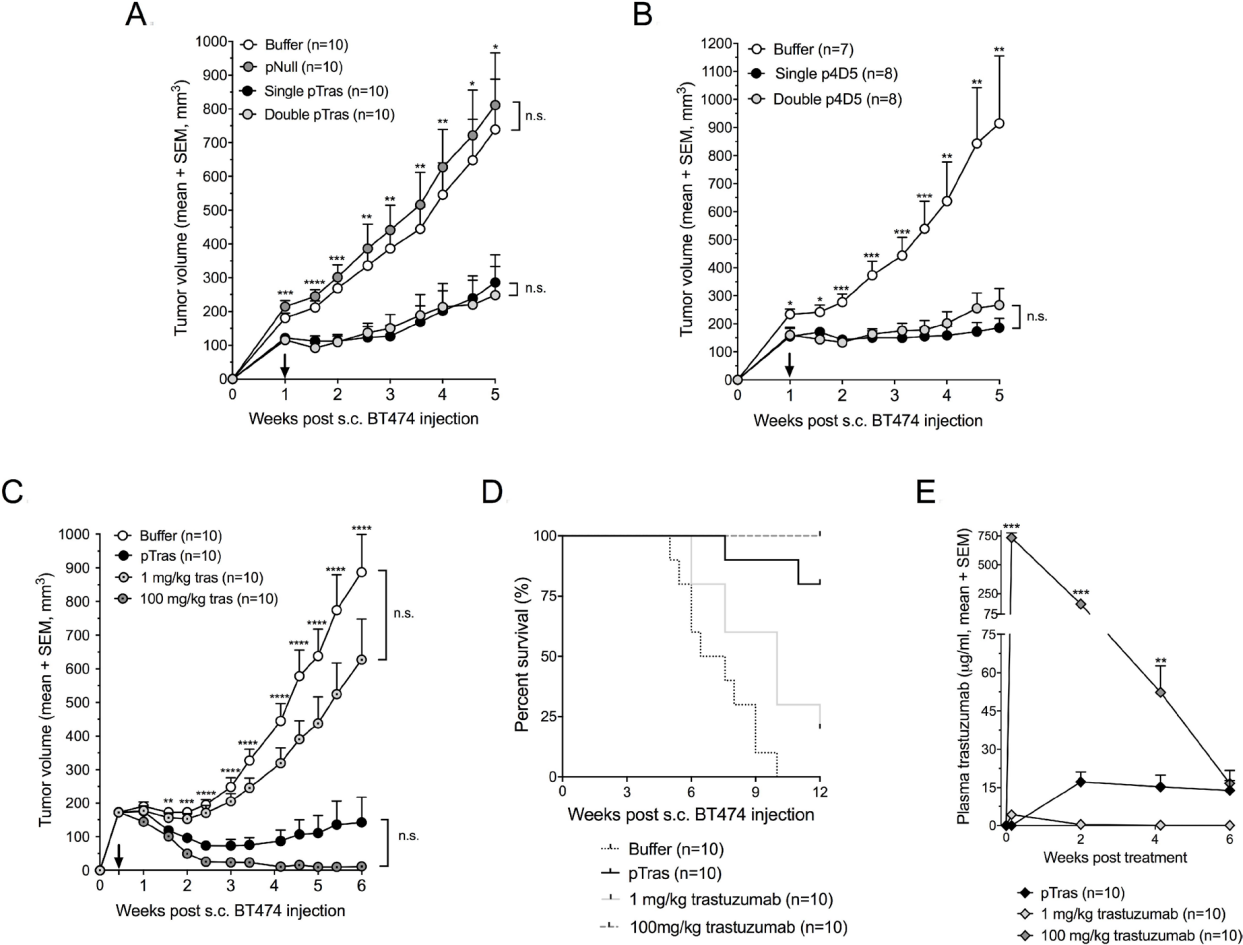
Efficacy and pharmacokinetics of DNA-based anti-HER2 antibodies in a mouse breast cancer model (**A**) Anti-tumor responses of prophylactic trastuzumab gene transfer in a BT474 breast cancer model in athymic nude mice. One week prior to subcutaneous (s.c.) injection of BT474 cells, mice received an intramuscular (i.m.) electrotransfer of buffer (D-PBS), pNull (an empty plasmid), or a 2 × 60 µg pCAG-tras dose (‘single pTras’ group). Half of the latter mice received an additional 2 × 60 µg pCAG-tras dose two weeks later, one week after s.c. tumor cell injection (‘double pTras’ group). Stars indicate the statistical difference between the pNull and single pTras group (^*^P < 0.05, ^**^P < 0.005, ^***^P < 0.001, ^****^P < 0.0001). (**B**) Anti-tumor responses of prophylactic 4D5 gene transfer in a BT474 breast cancer model. Experimental setup is identical to panel A, except no pNull group is included. Stars indicate the statistical difference between the buffer and single p4D5 group (^*^P < 0.05, ^**^P < 0.005, ^***^P < 0.001). (**C**) Anti-tumor responses of therapeutic trastuzumab gene transfer in a BT474 breast cancer model in athymic nude mice. Three days after s.c. injection of BT474 cells, mice received an i.m. electrotransfer of buffer (D-PBS), electrotransfer of four intramuscular 60 µg pCAG-tras injections, or an intraperitoneal (i.p.) injection of either 1 mg/kg or 100 mg/kg trastuzumab protein (Herceptin, Roche). Stars indicate the statistical difference between the buffer and pTras group (^**^P < 0.005, ^***^P < 0.001, ^****^P < 0.0001). Arrows indicate the timing of the second (A–B) or first treatment (C). (**D**) Survival curves of the different treatment groups (buffer, pTras, and 1 mg/kg or 100 mg/kg i.v. trastuzumab) until 12 weeks of follow-up, based on a 1000 mm^3^ tumor volume threshold. (**E**) Pharmacokinetic trastuzumab profile of the tumor-bearing athymic nude mice that received either an i.m. electrotransfer of pCAG-tras or an i.p. injection of 1 mg/kg or 100 mg/kg trastuzumab protein. Stars indicate the statistical difference between the pCAG-tras and 100 mg/kg trastuzumab group (^**^P < 0.005, ^***^P < 0.001). Tumor data were plotted as long as all groups were complete, i.e. no animals were sacrificed. Abbreviation; n.s.: not significant.

The *in vivo* efficacy of anti-HER2 mAb gene electrotransfer was evaluated in a BT474 breast cancer mouse model, either as a prophylactic or therapeutic. In the prophylactic setting, intramuscular pDNA electrotransfer was performed one week prior to subcutaneous tumor cell injection in athymic nude mice. To achieve higher mAb titers, the *tibialis anterior* and *gastrocnemius* were injected with 60 µg pDNA each. For one group of mice, this was the only treatment (‘single pDNA’). Another group of mice received a second identical pDNA dose two weeks later (‘double pDNA’), i.e. one week after BT474 cell injection. Prophylactic intramuscular pCAG-tras electrotransfer had a significant impact on tumor growth compared to buffer (D-PBS) or pNull (a plasmid without transgene expression cassette) (*P* < 0.001) (Figure 8A). After 12 weeks of follow-up, five complete tumor responses were observed: two out of ten in the single pCAG-tras group and three out of ten in the double pCAG-tras group. The additional pCAG-tras dose increased plasma trastuzumab concentrations (Supplementary Figure 1B), but failed to improve anti-tumor response. Among all mice that received pCAG-tras (*n* = 20), higher plasma trastuzumab did correspond to better anti-tumor responses, as exemplified by the negative correlation between plasma titer and tumor volume five weeks after tumor cell injection (*r* = -0.64, *P* < 0.005). This apparent contradiction is likely related to the variability in attained trastuzumab concentrations among both groups. Prophylactic pCAG-4D5 gave very similar findings, with single and double pDNA dosing demonstrating a significant and comparable anti-BT474 response (Figure 8B). The therapeutic setting focused on pCAG-tras. Based on the outcome of the prophylactic experiments, two modifications were made. First, to maximize mAb titers, mice received the four pDNA doses at once, rather than spread across two weeks, thereby targeting both legs with a total of 240 µg pCAG-tras. Second, the applied field strength was increased from 120 V/cm to 160 V/cm. Athymic nude mice typically have a larger muscle mass than BALB/c mice, which can lead to a higher tissue impedance and lower transfection efficiency, warranting adapted pulses. To assess how DNA-based mAb delivery compared to the conventional mAb protein administration, two groups of mice received a single i.p. dose of either 1 or 100 mg/kg clinical-grade trastuzumab protein. Treatment was initiated three days after BT474 cell injection when tumors were palpable. pCAG-tras had a significant impact on tumor growth, outperforming both buffer and 1 mg/kg trastuzumab (*P* < 0.001 from week 2 on). No significant difference was observed with 100 mg/kg trastuzumab (P between 0.12–0.70 from day 17 on). 1 mg/kg trastuzumab had no significant effect on tumor growth (Figure 8C). After 12 weeks of follow-up, pCAG-tras enabled stable disease in three out of ten, complete tumor response in three out of ten, and a lesion below 1 mm^3^ in one mouse. 1 mg/kg trastuzumab led to a complete tumor regression in one animal and no stable diseases. 100 mg/kg induced complete responses in eight out of ten, and a lesion below 1 mm^3^ in one mouse. Individual tumor volumes for each group are plotted in Supplementary Figure 1C. Anti-tumor responses were reflected in the survival curves of each group, using a tumor volume threshold of 1000 mm^3^. pCAG-tras outperformed buffer (*P* < 0.0001) and 1 mg/kg trastuzumab (*P* < 0.01) and showed no significant difference with 100 mg/kg trastuzumab (*P* = 0.146) (Figure 8D). The different trastuzumab plasma concentrations among treatment groups again illustrated the difference between DNA- and protein-based mAb administration (Figure 8E). After two weeks, trastuzumab was mostly cleared in the 1 mg/kg group, while expressed trastuzumab in the pCAG-tras group reached an average of 17 µg/ml – still significantly below the 160 µg/ml of the 100 mg/kg group. After six weeks, trastuzumab concentrations of pCAG-tras group were similar to those of the 100 mg/kg group (Figure 8E). Overall, no weight loss or other obvious signs of treatment toxicity were observed in any of the tumor experiments. Therapeutic pCAG-tras led to better anti-tumor responses than the prophylactic application. This is likely because of the higher trastuzumab concentrations in the former setting, which are associated with higher pDNA dosing and adjusted field strength. Overall, these results demonstrated that antibody gene electrotransfer enables therapeutically effective mAb titers, and can rival with the administration of milligrams of trastuzumab protein.

## DISCUSSION

Our study built pre-clinical proof of concept for antibody gene electrotransfer in oncology, and evaluated the impact of several underexplored parameters on the PK and efficacy of DNA-based trastuzumab or 4D5, including administration, plasmid backbone, expression cassette, and immunogenicity.

For pDNA administration, a robust intramuscular electroporation protocol was established using the guidance of a newly engineered triple-reporter cassette. In agreement with the literature [12, 13], both reporter and mAb expression was further increased using hyaluronidase pretreatment of the muscle. This enzyme is currently already applied in patients [14, 15], making it a potential adjuvant for clinical antibody gene electrotransfer. As plasmid backbone, we initially evaluated a ‘gene therapy-compatible’ version of the GTU, a plasmid designed by FIT Biotech and under clinical evaluation for HIV DNA vaccination [8]. gtGTU was engineered by the company to contain less immunostimulatory sequences than GTU (undisclosed data) while maintaining the mechanism of action. The backbone co-encodes the bovine papillomavirus type 1 E2 nuclear anchoring protein and simultaneously harbors multimeric E2 binding sites. This allows the expressed E2 to tether the gtGTU plasmid to chromatin, reportedly improving transgene expression via enhanced segregation and partitioning. In addition, E2 has been postulated to act as transcriptional activator for the gene of interest [7, 8]. Our findings, however, refute these claims. gtGTU consistently failed to provide benefit for *in vivo* reporter and mAb expression, and was abandoned in favor of a conventional plasmid. For expression cassette design, different promoters and configurations were explored. The use of tissue-specific promoters has been suggested to promote prolonged stable transgene expression [16, 17]. The muscle-specific ΔUSE previously showed a strength similar to potent ubiquitous promoters [9], and was therefore selected in the current study for evaluation. ΔUSE mAb expression, however, did significantly worse than CAG, limiting its relevance for said application. Using CAG, a dual mAb cassette system outperformed a single 2A-based cassette, despite the substantially larger size. The former construct subsequently emerged as our platform of choice for DNA-mediated antibody gene transfer. This was further supported by the high similarity between the in-house expressed trastuzumab and its clinical-grade counterpart.

Overall, the reporter data contributed to the design of a robust mAb expression system. At the same time, the current study illustrated the challenges in identifying favorable expression parameters. Moreover, irrespective of plasmid administration or engineering, the humoral immune response, i.e. ADAs, emerged as a critical hurdle for successful antibody gene transfer. This is in line with previous findings [18], but is characterized more in depth in the current study. Compared to protein delivery, DNA-based transfer clearly aggravated the ADA response. This observation is exploited in the field of DNA vaccines [19], but is not warranted for therapeutic gene transfer purposes. Two routes were thereto pursued: i) expressing trastuzumab in immune-compromised mice, and ii) gene transfer of the murine 4D5 IgG1 in immune-competent mice.

Both approaches allowed prolonged mAb expression, at least six to nine months after pDNA administration, and boosting of mAb expression by pDNA re-dosing. In the absence of an antibody immune response, trastuzumab and 4D5 demonstrated comparable *in vivo* expression, with plasma titers ranging between 1–15 µg/ml depending on the pDNA dose and construct engineering. The PK profile of expressed mAbs was in clear contrast to that of injected mAb protein, which, in accordance with the molecule’s half-life, was typically cleared a few weeks after administration.

Only few reports have reported the *in vivo* expression of anti-HER2 trastuzumab or 4D5. Most used viral vectors, which resulted in 30 to 120 µg/ml mAb plasma peak concentrations in mice [1, 20, 21]. Albeit our attained mAb levels were lower, significant anti-tumor responses were achieved in a breast cancer mouse model, including multiple complete regressions. DNA-based trastuzumab thereby outperformed a 1 mg/kg trastuzumab protein dose (≈ 20 µg), and was similar to 100 mg/kg trastuzumab (≈ 2 mg), a high dose in pre-clinical settings. As a reference, about 70% of studies on breast cancer mouse models apply trastuzumab dosing below 75 mg/kg [22]. Given the clear correlation between circulating mAb concentrations and anti-tumor responses, the efficacy of antibody gene transfer is expected to further benefit from advances that enhance transgene expression.

The generated PK and efficacy data illustrate the broad applicability of DNA-based antibody gene transfer. In pre-clinical R&D, it could serve as a straightforward tool to express and evaluate multiple DNA-based antibody leads *in vivo*, avoiding the laborious task of producing and purifying each lead. In the clinic, it could facilitate prophylactic, maintenance or combination treatments, thereby addressing the current hurdles with conventional mAb therapy. However, clinical translation of antibody gene transfer still faces several challenges and uncertainties, most notably biosafety (e.g. tumor and organ distribution, immunogenicity, and control of mAb expression) and efficacy (ability to attain therapeutic mAb titers in patients). Innovations in expression platform, administration and antibody technology are expected to address these challenges, and unlock the vast potential of antibody gene transfer.

In conclusion, our study provides critical insights in the design and application of DNA-based antibodies. Guided by reporter and construct engineering studies, we achieved robust and prolonged *in vivo* expression of two anti-HER2 mAbs, whose efficacy rivalled with high-dose mAb protein administration in a breast cancer model. Overall, the reported findings serve to advance antibody gene transfer in oncology and beyond.

## MATERIALS AND METHODS

### Cell lines and reagents

293F Freestyle suspension cells (purchased from Thermo Fisher Scientific in 2015) were maintained in FreeStyle 293 Expression Medium on a CO_2_-resistant orbital shaker (Thermo Fisher Scientific) in a 37° C humidified incubator at 8% CO_2_. The HER2+ human breast cancer cell line BT474 was a kind gift from Dr. Christos Sotiriou (Breast Cancer Translational Research Laboratory J.C. Heuson, Jules Bordet Insitute, Brussels, Belgium) in July 2014. Cell line identity was confirmed using short tandem repeat analysis at the Laboratory of Forensic Biomedical Sciences, KU Leuven, most recently in October 2017. Cells were grown in RPMI medium, supplemented with 10% heat-inactivated fetal bovine serum, 1% penicillin-streptomycin, and 1 mmol/L sodium pyruvate (Thermo Fisher Scientific), at 37° C in a humidified incubator at 5% CO_2_. Clinical-grade trastuzumab (Herceptin, 150 mg vial, H4426B02 lot) was kindly provided by Roche (Belgium), reconstituted in sterile milliQ H_2_O according to the instructions, aliquoted and stored at −80° C until use.

### Mice

Gene transfer and tumor experiments were performed in five to nine weeks old female mice with an approximate weight of 18–20 grams. BALB/c mice (BALB/cAnNCrl) were bred at the KU Leuven Animal Research Center or purchased at Janvier (France). Athymic nude mice (hsd: athymic nude-foxn1^nu^) were purchased at Envigo (The Netherlands). C57BL/6J RAG2^*−/−*γ^c^*−/−*^ mice were purchased from the division Translational Research in Gastrointestinal Disorders (Department of Chronic Diseases, Metabolism and Ageing) at KU Leuven. Plasma was collected via retro-orbital bleeding, processed and stored at −20° C until analysis. All animal experiments were approved by the KU Leuven Animal Ethics Committee (project P163/2013).

### Reporter- and mAb-encoding plasmids

Reporter or mAb cDNA sequences were cloned into the gtGTU plasmid (developed and provided by FIT Biotech under research agreement) or a conventional plasmid. Both plasmids include an ampicillin-resistance gene and pUC origin of replication, and are identical except for the presence of the E2 gene cassette and multimeric E2 binding sites (Figure 1). In gtGTU, 10 copies of the E2 binding sites are located directly upfront of the promoter. The E2 expression cassette, driven by a Rous sarcoma virus 5´ long terminal repeat promoter, is placed in tandem of the reporter or antibody cassette [7, 8]. The Kozak sequence preceded the start codon of each transgene followed by an appropriate signal peptide. cDNA sequences of the murine anti-HER2 4D5 (IgG1) and of humanized trastuzumab (IgG1) were derived from the literature [5]. Transgene sequences were codon-optimized for mouse and synthesized by Genewiz, and cloned into the plasmid expression cassettes by Icosagen. Synthesis and cloning were verified via sequencing and restriction analyses. *In vitro* transgene expression was evaluated by ELISA, SDS-PAGE or Western blot (data not shown). pDNA was produced in *E. coli*, purified using the NucleoBond Xtra Maxi EF kit (Machery - Nagel) according to the manufacturer’s instructions, and eluted in sterile D-PBS (no magnesium, no calcium, 14190144, Thermo Fisher Scientific). Plasmid purity and integrity was assessed via spectrophotometry and agarose gel electrophoresis.

### Intramuscular pDNA electrotransfer in mice

pDNA administration was done in the *tibialis anterior* muscle, unless stated otherwise. The site of delivery was prepared using depilatory product (Veet, Reckitt Benckiser), at least one day prior to pDNA injection. For antibody gene electrotransfer, muscles were injected with 40 µl of 0.4 U/µl hyaluronidase from bovine testes (H3506, Sigma, reconstituted in sterile saline**)**, approximately one hour prior to pDNA electrotransfer. Intramuscular injection of 30 µl of pDNA, formulated in sterile D-PBS at the indicated amounts, was immediately followed by *in situ* electroporation using the NEPA21 Electroporator (Sonidel) with CUY650P5 tweezer electrodes (Sonidel) at a fixed width of 5 mm. Signa Electrode Gel (Parker Laboratories) was applied to the muscle and electrodes to decrease tissue impedance below 0.4 kiloohm. Pulse delivery was verified using the NEPA21 readout.

### *In vivo* reporter imaging

eGFP expression was visualized using a microscope with a fluorescent light source (Leica MZ10F). fluc expression was quantified by non-invasive bioluminescence imaging (IVIS Spectrum, Perkin Elmer) at the molecular imaging for Small Animals Center (moSAIC) at KU Leuven. Mice were sedated with isoflurane inhalation and subcutaneously injected with 126 mg/kg D-luciferin substrate (Promega) at 15 mg/ml in sterile D-PBS (14190144, Thermo Fisher Scientific). Total radiance (p/s, photons per second) was collected at fluc signal plateau. *G*luc in plasma was analyzed using a FLUOstar Omega Microplate Reader (BMG Labtech) in black 96-well plates. Per well, 100 µl of 100 µM coelenterazine (Nanolight Technology, Pinetop, AZ) was added to 5 µl plasma via automated injection. Data was analyzed with MARS Software (BMG Labtech). Counts (RLU, relative light unit) 1 sec after injection of the substrate were used for analyses. Readout was corrected for the pretreatment plasma count.

### *In vitro* mAb production and purification

Trastuzumab and 4D5 proteins were produced *in vitro* in 293F cells and purified from the supernatant. Transfection was done using pCAG-tras or pCAG-4D5 with X-tremeGENE HP DNA Transfection Reagent (Roche) in Freestyle media (Thermo Fisher Scientific), following the manufacturer’s protocol. Five days after transfection, cells and media were collected. Supernatant was obtained via centrifugation and 0.2 µm filtration and stored at −20° C. Subsequent purification of the expressed mAbs was done on ÄKTAprime plus (GE Healthcare Life Sciences), using a 1 ml pre-packed column with the Protein A affinity resin Amsphere A3 (JSR Life Sciences) according to the manufacturer’s protocol. Following elution with 100 mM sodium acetate pH 3.5 and neutralisation with 1M Tris pH 9, fractions were pooled and dialysed to 20 mM sodium phosphate, 150 mM NaCl pH 7.5, aliquoted and stored at −80° C. Batches of purified mAb were evaluated for consistency on a HER2-coated ELISA and by SDS-PAGE (under non-reducing and 2-mercapto-ethanol reducing conditions). For the latter, 2.5 µg of mAb was formulated in NuPAGE LDS Sample Buffer, loaded on a 10-well NuPage Bis-Tris 4–12% Protein Gel and run in NuPage MES SDS Running Buffer in a XCell SureLock Mini-Cell Electrophoresis System (Thermo Fisher Scientific), according to the manufacturer’s instructions. Novex Sharp Pre-stained Protein Standard (LC5800, Thermo Fisher Scientific) was used as marker. Pictures of the Coommasie blue-stained gel were taken with the ChemiDoc MP System (Bio-Rad) and processed using Image Lab Software 6.0 (Bio-Rad).

### ELISA for mAb quantification

Trastuzumab concentrations were quantified with a HER2-coated SHIKARI Q-TRAS ELISA (Matriks Biotek), according to the manufacturer’s instructions. A dedicated 4D5 ELISA was developed in-house. 96-well plates were coated overnight at 4° C with 500 ng/ml human HER2 (10004-H08H, Sino Biologicals) in PBS. Plates were blocked with 1% BSA in PBS for two hours at RT. Samples were diluted in PTAE (PBS 0.1% BSA, 0.002% Tween 80, 5 mM EDTA) and incubated on the blocked HER2-coated plates for one hour at RT. Serial two-fold dilutions of purified 4D5, with concentrations ranging between 60 and 0.94 ng/ml, were used as calibration curve. Detection of the captured 4D5 was done with goat anti-mouse IgG – HRP (GAM/IgG/PO, Novo Nordisk, 1:10000 dilution in PTA) and incubated for one hour at RT. Each incubation step was preceded by a washing step with PBS 0.05% Tween 20. Plates were developed for 30–45 minutes using o-phenylenediamine and H_2_O_2_ in citrate buffer. The reaction was stopped with 4 M H_2_SO_4_. Absorption was measured at 490 nm using an ELx808 Absorbance Microplate Reader (BioTek Instruments). Sample concentrations were calculated based on the 4D5 calibration curve using a non-linear regression fit (GraphPad Prism 7.0).

### ELISA for ADA detection

The presence of antibodies against the *in vivo* expressed mAb was assessed via a drug-sensitive bridging or a drug-tolerant affinity capture elution (ACE) ELISA setup. The bridging assay was performed as follows. 96-well plates were coated overnight at 4° C with 1 µg/ml trastuzumab (Herceptin, Roche) or 500 ng/ml purified 4D5 in PBS, and blocked with 1% BSA in PBS for two hours at RT. Plasma samples were diluted in PTAE and incubated on the blocked mAb-coated plates for one hour at RT. Detection of captured antibodies was done with biotinylated trastuzumab or 4D5 (EZ-Link Sulfo-NHS-LC-Biotin, Thermo Fisher Scientific), respectively, at a concentration of 500 ng/ml in PTA, followed by 30 minutes incubation with streptavidin-poly HRP (M2032, Sanquin) 1:10000 in PTA. Each incubation step was preceded by a washing step with PBS + 0.05% Tween 20. Plates were developed for 30–45 minutes using o-phenylenediamine and H_2_O_2_ in citrate buffer. The reaction was stopped with 4 M H_2_SO_4_. Absorption was measured at 490 nm using an ELx808 Absorbance Microplate Reader (BioTek Instruments). The drug-tolerant ACE assay was set up as previously described [23].

### *In vitro* biological activity of the expressed mAbs

The activity of trastuzumab and 4D5, purified from pCAG-tras or pCAG-4D5 transfected 293F cell supernatants, was evaluated in a WST-8 BT474 cell viability assay (Cell Counting Kit, Dojindo). 10000 BT474 cells were seeded per well in a 96-well plate, followed by the immediate application of serial dilutions of the mAbs, with concentrations ranging from 500 to 10 ng/ml. 120 hours after mAb administration, WST-8 was added and incubated up to 5 hours at 37° C. Absorptions were measured at 450 nm using an ELx808 Absorbance Microplate Reader (BioTek Instruments), and normalized between cell controls of medium and no-cell wells of medium. IC_50_ values were derived from a non-linear fitted sigmoidal dose-response curve (GraphPad Prism 7.0).

### Breast cancer mouse model

Five to six weeks old female athymic nude mice were subcutaneously injected in the flank with 5 × 10^6^ BT474 cells in 150 µl RPMI medium (Thermo Fisher Scientific) at 4 mg/ml matrigel (356235, BD Biosciences). To assure cell growth of these estrogen-dependent tumor cells, mice received a subcutaneous injection of 100 µg 17β-estradiol valerate (E1631, Sigma) in 50 µl arachnoid oil (Vandemoortele) starting one day before cell engraftment and continued weekly until experiment completion. Tumor volume was calculated using the formula: *a x b*^*2*^ *x 0.5*, with *a* being the tumor length and *b* the width. Measurements were done in duplicate using a digital caliper (5OLD).

### Statistics

Statistical analyses and figure drawing were done using GraphPad Prism 7.0 (GraphPad Software). Data were presented as mean ± standard error of mean (SEM) and analyzed using the Student’s *t*-test, ANOVA and Tukey’s multiple comparison test, or Pearson’s correlation coefficient test. Survival curves were compared using Log-rank (Mantel-Cox) test. Two-sided *P* values below 0.05 were considered significant.

## SUPPLEMENTARY MATERIALS FIGURE



## Abbreviations

ACE: affinity capture elution
ADA: anti-drug antibodies
eGFP: enhanced green fluorescent protein
fluc: firefly luciferase
GEF: triple-reporter expression cassette with *G*luc, eGFP and fluc
*G*luc: *Gaussia* luciferase
gtGTU: gene therapy-compatible Gene Transport Unit
HC: heavy chain
HER2: human epidermal growth factor receptor 2
hyal: hyaluronidase
i.m.: intramusuclar
i.p.: intraperitoneal
i.v.: intravenous
IC_50_: half-maximal inhibitory concentration
LC: light chain
mAb: monoclonal antibody
p/s: photons per second
pDNA: plasmid DNA
RLU: relative light unit
s.c.: subcutaneous
