# Supplementary material for: Prolonged in vivo expression and anti-tumor response of DNA-based anti-HER2 antibodies
Source: Oncotarget. 2018 Feb 6;9(17):13623–36. doi: 10.18632/oncotarget.24426 (PMC5862603; doi:10.18632/oncotarget.24426)
Supplement: Supplementary file 1 [file oncotarget-09-13623-s001.pdf]

Prolonged *in vivo* expression and anti-tumor response of DNA-based anti-HER2 antibodies

SUPPLEMENTARY MATERIALS

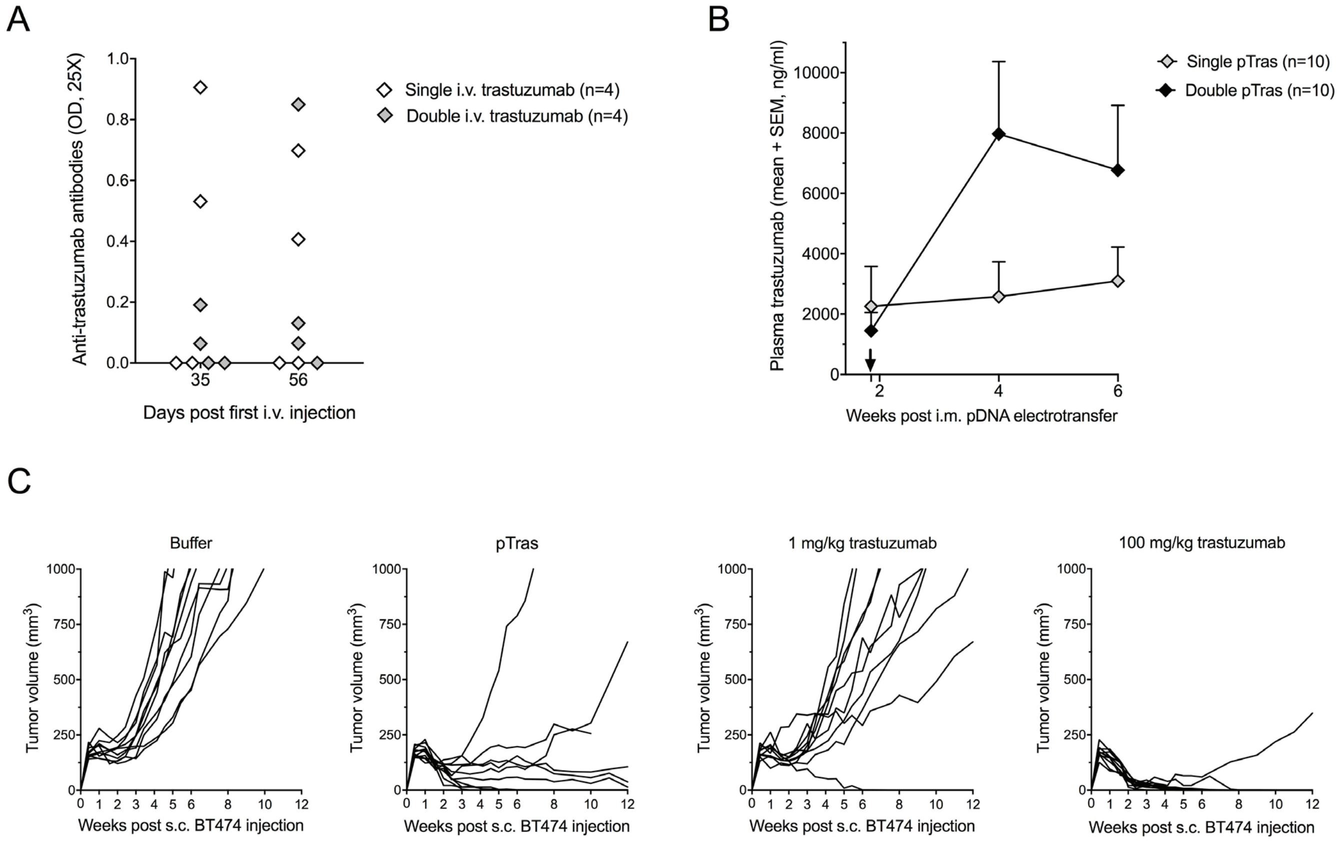

Supplementary Figure 1: (**A**) Plasma anti-trastuzumab antibodies, measured with a drug-sensitive ELISA, in BALB/c mice that received either one or two i.v. injections of 0.2 mg/kg trastuzumab protein. (**B**) Plasma trastuzumab concentrations of the 'single pTras' and 'double pTras' groups. The latter received an additional intramuscular (i.m.) pCAG-tras dose two weeks after the first dose. (**C**) pCAG-tras therapeutic experiment in athymic nude mice: individual tumor volumes of the different treatment groups. Abbreviations; i.v.: intravenous.
